# Intensification of Enzymatic Sorbityl Laurate Production in Dissolved and Neat Systems under Conventional and Microwave Heating

**DOI:** 10.1021/acsomega.3c10004

**Published:** 2024-04-01

**Authors:** André Delavault, Oleksandra Opochenska, Sonja Schönrock, Rebecca Hollenbach, Katrin Ochsenreither, Christoph Syldatk

**Affiliations:** † Technical Biology, Institute of Process Engineering in Life Sciences II, Karlsruhe Institute of Technology, Karlsruhe 76131, Germany; ‡ Biotechnological Conversion, Technikum Laubholz GmbH, Göppingen 73033, Germany

## Abstract

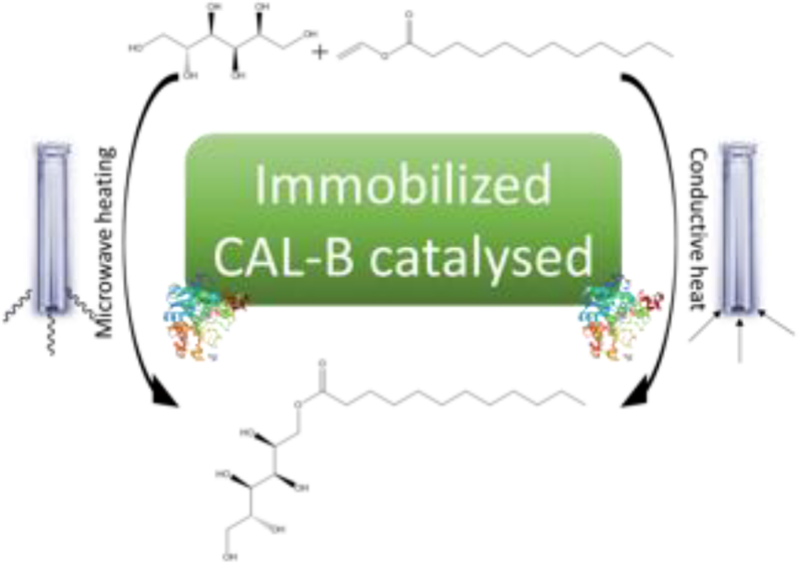

Glycolipids such as sugar alcohol esters have been demonstrated to be relevant for numerous applications across various domains of specialty. The use of organic solvents and, more recently, deep eutectic solvents (DESs) to mediate lipase-supported bioconversions is gaining potential for industrial application. However, many challenges and limitations remain such as extensive time of production and relatively low productivities among others, which must be solved to strengthen such a biocatalytic process in industry. In this context, this study focuses on the intensification of sorbityl laurate production, as a model biocatalyzed reaction using Novozym 435, investigating the relevance of temperature, heating method, and solvent system. By increasing the reaction temperature from 50 to 90 °C, the space-time yield and product yield were considerably enhanced for reactions in DES and the organic solvent 2M2B, irrespective of the heating method (conventional or microwave heating). However, positive effects in 2M2B were more pronounced with conventional heating as 98% conversion yield was reached within 90 min at 90 °C, equating thus to a nearly 4-fold increase in performance yielding 118.0 ± 3.6 g/(L·h) productivity. With DES, the overall yield and space-time yield were lower with both heating methods. However, microwave heating enabled a 2-fold increase in both performance parameters when the reaction temperature was increased from 50 to 90 °C. Compared to conventional heating, a 7-fold increase in space-time yield at 50 °C and a 16-fold increase at 90 °C were achieved in DES by microwave heating. Furthermore, microwave irradiation enabled the usage of a neat, solvent-free system, representing an initial proof of concept with productivities of up to 13.3 ± 2.3 g/(L·h).

## Introduction

Surfactants are a class of specialty compounds present in our daily lives and are applied in a wide spectrum of products ranging from household chemicals to agriculture.^1^ Most of their industrial production is made possible owing to nonrenewable petroleum-based substrates or renewable oleo chemical-based substrates followed by various chemical modifications.^2^ Many industries discharge their liquid waste, containing a wide range of surfactants, to wastewater treatment facilities. Once used, most surfactants of synthetic nature are not biodegraded and enter the water bodies where their persistence results in harmful accumulation. Toxicity of surfactants has aroused a worldwide alert leading to various regulations on their usage and disposal.^3^ In response, increased efforts are devoted to developing “green” surfactants, which are not only safer for customers and the environment but also sustainable regarding their production.^4^ Thus, the design of processes enabling the production of so-called “biosurfactants” makes sense from this perspective of sustainability. To date, significant progress in biotechnology, stricter regulatory requirements, and industrial expectations regarding the toxicity and costs of newly synthesized surfactants have delivered additional stimulus for the development of biosurfactants that are of great potential or currently used in various industrial applications (e.g., food or cosmetics industries among others).^5^

In this context, biocatalysis offers both economic and environmental advantages over chemocatalytic methods, which can be even further strengthened via the immobilization and reuse strategy of the biocatalyst.^6^ Indeed, enzymes are produced from inexpensive renewable resources and are biodegradable, thus respecting intrinsically fundamental principles of green chemistry and sustainable developments.^7^ In contrast, organometallic catalysts demand metals like rhodium for asymmetric transformations, which is one of the scarcest metals on earth.^8^ Thus, in comparison, synthesis using enzymes is often cheaper, more facile, and amenable.

Previous work has demonstrated the enzymatic production of sorbityl laurate (SL), a useful surfactant belonging to the glycolipid subfamily and may be included in the composition of certain oil-in-water emulsifier potentially suitable for cosmetic applications.^9^ The synthesis was rendered possible in a so-called “2-in-1” deep eutectic system (DES), made of choline chloride in combination with sorbitol, the latter being implicated simultaneously in the bioconversion. This principle was first reported by Siebenhaller et al.^10^ and conjointly reviewed in an article by Pätzold et al.^11^ However, the performance of this specific proof of concept, using mainly glucose as an acyl acceptor, was exceptionally poor (∼4% yield). Recently, the conversion yield of this process could be increased 7-fold compared to the original proof of concept by using better acyl acceptors, such as sugar alcohols, and optimizing the reaction parameters such as water content, substrate concentration, and biocatalyst load among others. Scalability of the process was demonstrated as well, but, in the meantime, mass transfer was *inter alia* highlighted as a potential limitation to the performance of the batch-process during the scale-up to a stirred tank reactor. Indeed, not only an overall 2-fold loss of titer but also a considerable loss of specific reaction rate was observed after only 4 h of reaction, ending up in an overall lower reaction rate.^12^ Nonetheless, the intrinsic advantages of low-temperature transition mixtures, like DESs, such as their straightforward production method, amenable use, low price, and relative harmlessness have to be taken into account.^13−16^ Therefore, it is of interest to make these media competitive to standard organic solvents to gain applicability and industrial interest as they have also more room for process intensification owing to their novelty in the field of biocatalysis.^17^

Process intensification, an ever-growing field that aims at tackling developmental problems by means of modern engineering to continuously increase efficiency of chemical and biochemical processes, comes then into play.^18^ In that regard, utilization of microwave (MW) heating has been reported to dramatically enhance performances of numerous chemical reactions, extractions, and biomass valorization processes while decreasing equipment size to output ratio, waste production, and energy consumption, which fits perfectly the aims of process intensification.^19^ MW technology is based on dielectric heating. In conventional heating (CH), on the other hand, the energy is transported via the thermal conductivity of the reaction medium. Consequently, higher and inhomogeneous heating of the reactor walls than the reaction bulk can be caused, which is in opposition to MW technology that should allow an effective and internalized heating.^20^ However, MW is rather scarcely represented in the literature and is still a niche topic for sugar ester production specifically.^21^ It seemed to be a promising tool to intensify biocatalyzed reactions as previous reports outline synergistic effects between the resulting dielectric heating and biocatalysis.^22^ Indeed, the reaction rates and overall yields of rather slow enzyme-catalyzed reactions have been tremendously increased.^23−26^

**Figure 1:**
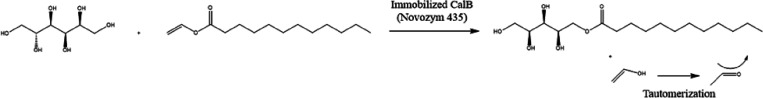
Reaction scheme of the model enzymatic reaction producing SL by the use of D-sorbitol as an acyl acceptor and vinyl laurate as an acyl donor. Reaction is made irreversible by formation of ethenol, which tautomerizes to acetaldehyde, forcing thus the reaction in the direct sense according to Le Chatelier′s thermodynamic principle.

In the present follow-up study, we aim at investigating the relevance of organic solvents and solvent-free reactions compared to DES and MW irradiation as alternatives to conventional heating to enhance the performances of our model biocatalyzed glycolipid production (Figure 1). Comparisons between the different solvent systems and heating methods in overall yields and productivities are bringing thus nuance to the promotion of DESs as green media for biocatalysis.

## Experimental Section

### Materials

Vinyl laurate was purchased from Tokyo Chemical Industry Co., Ltd. (TCI Europe, Belgium). Lipase formulation Novozym 435 (9000 PLU/g) was given by Novozymes (Denmark). All other chemicals and solvents were purchased from either Carl Roth GmbH & Co. KG (Karlsruhe, Germany) or Sigma-Aldrich Chemie GmbH (Taufkirchen, Germany), if not stated otherwise.

### DES Preparation and Control

The sorbitol and choline chloride-based DES (mole ratio 1:1), dubbed SDES, was prepared and validated according to the procedures described by both Dai et al. and Hayyan et al.^27,28^ Its water content was fixed at 5 wt % and controlled by Karl Fischer titration using a TritoLine 7500 KF trace from SI Analytics (Mainz, Germany) at 20 °C in combination with Aquastar CombiCoulomat fritless (Merck Millipore, Darmstadt, Germany) as a reagent.

### Solvent Screening in CH

In a G10 Anton Paar vessel (Ostfildern, Germany) were introduced subsequently 3 mL of solvent 2-methyl-2-butanol (2M2B), ethyl-L-lactate (EL), acetonitrile (MeCN), methyl ethyl ketone (MEK), acetone (ACE), or sorbitol-based DES (SDES) and then vinyl laurate (195 μL, 0.75 mmol, 0.5 M), sorbitol (not added with SDES, 273 mg, 0.75 mmol, 0.5 M), and 30 mg of enzyme formulation (20 g/L). The tubes containing the reaction mixture were provided with a magnetic stirring bar and were agitated at 600 rpm and heated with a water/glycerin bath (50/50 v:v) at 50 °C on a Hei-PLATE Mix ′n′ Heat Core+ (Heidelberg, Germany). To get a triplicate for each measure, three tubes were collected for each time point at 0.5, 4, 8, 24, 28, 32, and 48 h.

Yields of SL in the respective solvent systems were calculated as follows:Reaction carried out in 2M2B or other organic solvents:
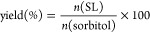
1Reaction carried out with SDES:
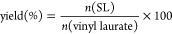
2For any solvent system:
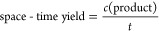
3where *n* is the number of moles, *c* is the mass concentration in g/L, and *t* is the operating time in h.

### Influence of Biocatalyst, Sorbitol, and Vinyl Laurate Concentrations in 2M2B

To examine the optimal concentration of lipase formulation, different concentrations of Novozym 435 (10, 20, 30, 40, 50, and 60 g/L) were tested without varying any other reaction parameter. To address the optimal substrate concentrations for the reaction, different vinyl laurate and sorbitol concentrations (0.25, 0.5, 0.75, 1, and 1.25 M) were tested independently from each other while all other reaction conditions were kept constant.

### Microwave Irradiation, Influence of Temperature, and Stirring Speed

In a 10 mL Microwave G6 tube were introduced subsequently 3 mL of 2M2B, vinyl laurate (585 μL, 510 mg, 2.25 mmol, 0.75 M), sorbitol (273 mg, 1.5 mmol, 0.5 M), and 60 mg of Novozym 435 (20 g/L). The tubes containing the reaction mixture were placed in a Monowave 400 (Anton Paar, Ostfildern, Germany) and subjected under various magnetic stirring speeds (200, 400, 600, 800, and 1000 rpm) and at temperatures of 50, 60 70, 80, 90, and 100 °C.

### Solvent-Free Reactions

In a 10 mL Microwave G6 tube was introduced 5.2, 2.1, 1.6, or 1 mmol of sorbitol combined with an adequate amount of vinyl laurate or lauric acid to finally achieve 1:2, 1:5, 1:7, or 1:11 sorbitol/fatty acid or ester molar ratios to keep a constant operational volume (*V*_R_ = 3 mL considering ρ_sorbitol_ = 1.49 g/mL, ρ_vinyl laurate_ = 0.87 g/mL, and ρ_lauric acid_= 0.88 g/mL). The mixture was completed by addition of 60 mg of Novozym 435 (20 g/L). The tubes containing the reaction mixture were placed in the Monowave 400 (Anton Paar, Ostfildern, Germany) provided with a magnetic bar and stirred at 400 rpm while being heated as fast as possible to 90 °C.

### Enzyme Recycling

The reusability of Novozym 435 was tested in 2M2B under optimized conditions (0.25 M sorbitol, 0.75 M vinyl laurate, and 20 g/L Novozym 435) at 50 and 90 °C using MW and CH. After 90 min of synthesis, the supernatant was discarded. The enzyme was washed with 3 × 3 mL of warm (75 °C) 2M2B and vortexed for 30 s to remove unreacted substrates. Then, the enzyme was provided with fresh media and substrates. The residual activity in the following cycles was calculated as a percentage of the conversion obtained in the first cycle.

### Sample Preparation and HPLC-ELSD Quantification Method

Tubes containing organic solvents were, postreaction, subsequently dry-evaporated on a SpeedVac centrifugal evaporator (H. Saur Laborbedarf, Reutlingen, Germany) and resuspended in 1 mL of chloroform:methanol (75:25 v/v); other tubes containing DES were prepared and analyzed via HPLC-ELSD as previously described by Delavault et al.^12^ The calibration curve used for quantification of SL is shown in Figure S1, and the SL standard produced in-house is shown in Figure S2.

### Data Treatment and Statistical Analysis

OriginPro software 10.0 [version 2023] (OriginLab Corporation, Northampton, MA, USA) was used for raw data treatment and statistical analysis. Results are presented as mean ± standard deviation (*n* = 3). Statistical analysis was performed by one-way ANOVA and Tukey test, and results were considered significant if the *p*-value was <0.05 if not stated otherwise.

## Results and Discussion

In the following sections, we report the investigation of several solvent systems under CH to produce SL alongside the investigation of the impact of several synthesis factors in 2M2B assisted by both CH and MW. Following the product formation over time also allowed optimization of reaction time, enzyme, and substrates concentrations. Subsequently, we transferred the optimized process to the MW synthesizer and compared the performances at different temperatures to those obtained with CH. Finally, we studied the effect of reusing the biocatalyst not only over three batch cycles but also across two different temperatures as well as two different reaction media. By comparing different heating and solvent systems for a defined biocatalyzed reaction, the influence of heat and mass transfer can be elucidated.

### Screening of Solvents under Conventional Heating

**Figure 2:**
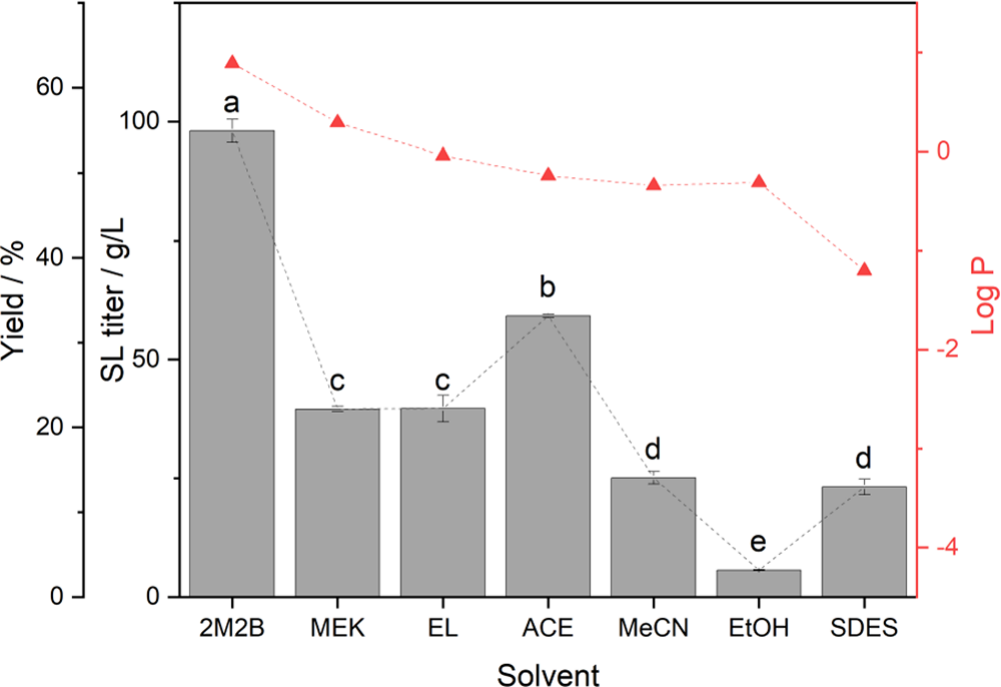
Comparison of SL titers and yields with nonoptimized conditions in several solvent systems: 2-methyl-2-butanol (2M2B), methyl ethyl ketone (MEK), ethyl-L-lactate (EL), acetone (ACE), acetonitrile (MeCN), ethanol (EtOH), and sorbitol-based DES (SDES), correlated to their respective log *P* (values of logarithm of the partition coefficient of octanol and water according to Laane et al.^32^ and Nian et al.^33^). A triplicate was done for each screened solvent after 48 h of reaction at 50 °C. a–e show statistically significant differences (*p* < 0.05).

Organic solvents have been used as reaction media for the synthesis of sugar alcohol esters in several studies. For example, sugar monoesters have been successfully produced from solid carbohydrates and vinylated fatty acids with high selectivity and high yield in solvents such as acetonitrile, acetone, and 2M2B.^29^ It was also reported that the latter is rather compatible with food and pharmaceutical applications for the preparation of monoesters from different sugars and sugar alcohols.^30^ Therefore, the suitability of several polar organic solvents as well as one DES consisting of sorbitol and choline chloride (SDES), enabling the “2-in1”-concept, to serve as reaction media was compared with respect to product titer and yield. Figure 2 shows that the highest performance was obtained in 2M2B using lipase B from the *Candida antarctica* (CalB) formulation, Novozym 435. Without optimization in 2M2B, almost ∼100 g/L was reached, which corresponds to a relatively high specific productivity at the tube scale of 287 μmol/g_biocatalyst_/h. In comparison, the DES system shows a lower performance after 48 h (∼20% yield) but displays a relatively low vapor pressure with minimized quality-of-air impairment. Indeed, the used DES system in this study, SDES, is composed of 5 wt % water and displays a water activity value of 0.077 at 50 °C, while 2M2B displays a vapor pressure of 12 mmHg at 20 °C. In consequence, SDES presents strongly bound water molecules that also compose its corresponding vapor, while 2M2B tends to be more volatile and suggests a greater hazard for the operators.^12,31^

In acetone, 60 g/L titer was realized, which is significantly higher than other recent and green solvent alternatives such as ethyl-L-lactate (EL), methyl ethyl ketone (MEK), and ethanol. Paradoxically, both alcohol solvents employed here showed drastic differences, as 2M2B, a tertiary alcohol, is about 10 times more effective than ethanol, a primary alcohol, to mediate the biocatalysis. With the latter, the lowest titer in SL was reached, which could be attributed to a greater destabilization of the enzyme′s tertiary structure or a disruption of the enzyme′s essential water shell as ethanol is highly hydrophilic and water is miscible with a log *P* value of −0.31 compared to 0.89 for 2M2B. In addition, to explain such inferior yield, it is likely that ethanol reacted with vinyl laurate under the action of the immobilized lipase to form ethyl laurate as similar reactions were previously reported in the literature.^34,35^

One of the main roles for solvents in the enzymatic reaction is to solubilize both substrates and retain the enzyme activity. Sorbitol solubility is very high in water and in many alcohols due to formation of hydrogen bonds,^36^ but it is insoluble in most nonpolar and aprotic solvents. It is also used as a hydrogen bond donor (HBD) in the formation of SDES and allows production of SL according to the “2-in-1” principle previously described. Vinyl laurate is slightly soluble in water (1 g/L at 20 °C) and well soluble in most alcohols. The log *P* parameter describes the hydrophobicity of the solvent, which explains its tendency to partition between phases with different polarities. 2M2B is considered a polar solvent with a log *P* value of 0.89, and ethanol, acetone, acetonitrile, and ethyl-L-lactate are also all polar with log *P* values of −0.31, −0.16, −0.33, and −0.18, respectively. It was shown that the active site of the *C. antarctica* lipase remains stable in nonpolar solvents (log *P* > 4), while polar solvents (log *P* < 2) can interact with the active site and break the hydrogen bond between the amino acid residues important for lipase activity.^37^ Solvents being partly water-miscible (log *P* between 2 and 0) show less impact as compared to the fully water-miscible ones (log *P* between 0 and −2.5) on the performance of the enzyme. As in our case, a rather polar and partly water miscible solvent such as 2M2B displayed fitting characteristics for process intensification. On the other hand, the choline chloride and sugar alcohol-based DES possesses a low log *P*,^33^ even lower than that of EtOH; however, counterintuitively, higher titers were observed, which can be attributed to the enzyme-stabilizing effects described for DESs in the literature.^38^ In addition, it is worth noting that the enzyme doubtlessly benefits from its acrylic resin carrier and thus tends to be less susceptible to the solvation effects of the various solvents that have been tested. Indeed, complete loss of activity potentially due to disruption of the tertiary structure should have been observed but activity can still be seen in solvents possessing log *P* values close to zero, as the immobilized lipase is less sensitive overall to experimental conditions.^6^ Altogether, 2M2B seemed to be a more promising option for further factor optimizations.

### Factor Optimization in 2M2B under Conventional Heating

**Figure 3:**
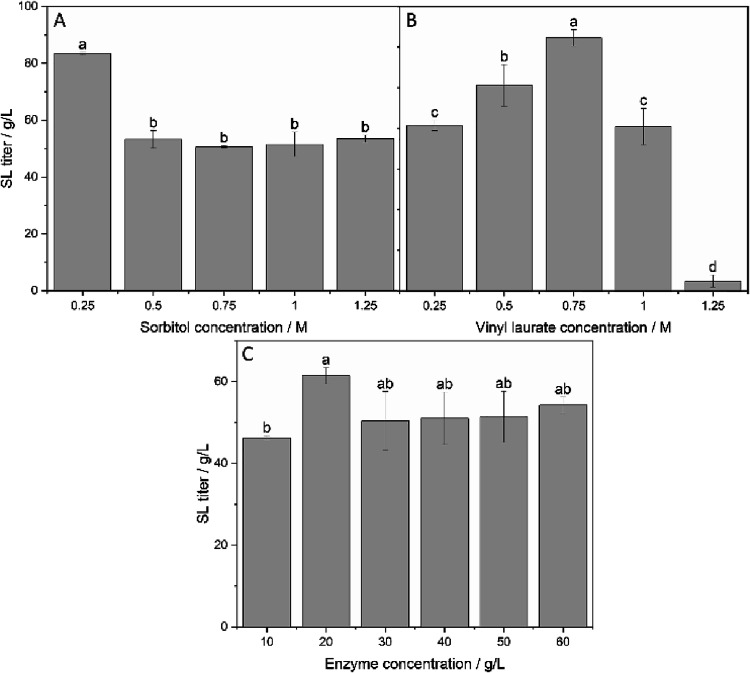
Novozym 435-catalyzed transesterification of sorbitol and vinyl laurate in 2M2B at 50 °C after 8 h of reaction. Influence of (A) sorbitol concentration and (B) vinyl laurate concentration and (C) effect of enzyme dosage. a–d show statistically significant differences at a 0.05 significance level of the mean values obtained from three independent experiments. Parameters were varied one at a time, keeping the other factors constant as follows: (A) 0.5 M (195 μL, 0.75 mmol) vinyl laurate and 50 g/L Novozym 435, (B) 0.25 M (136 mg, 0.375 mmol) sorbitol and 50 g/L Novozym 435, and (C) 0.25 M sorbitol and 0.75 M (292.5 μL, 1.125 mmol) vinyl laurate.

Factors impacting the reaction (i.e., substrate concentrations and enzyme dosage) have been investigated to find the optimum of each condition. It was thus determined that the optimal values were 20 g/L enzyme, 0.75 M vinyl laurate, and 0.25 M sorbitol when the reaction was carried out at 50 °C (Figure 3). In our study, prolonged incubation time seems to lead to product degradation as the titer decreased in 2M2B after 8 h of reaction (Figure S3). This observation could be explained by the formation of water due to the hydrolysis reaction of vinyl laurate leading to lauric acid reacting with sorbitol through the action of the lipase and thus generating even more water, in addition to the enzymatic hydrolysis of vinyl laurate.^39^

In previous work from our group, the reaction in SDES was similarly optimized with one factor at a time (OFAT) method.^12^ Thus, in light of the present results obtained using 2M2B, the optimization and intensification strategies were pointed toward the latter as a reference for enzymatic synthesis of sugar-based surfactants and is, as other alcohols, regarded as a relatively safer and sustainable organic solvent.^40,41^ The results displayed in Figure 3A suggest that using 0.25 M sorbitol induces a higher titer of SL. Drastic differences can be observed in Figure 3B as 0.75 M vinyl laurate induces a 1.5-fold increase in titer compared to 0.25 M and a 10-fold increase in comparison to 1.25 M. This suggests that upon a range of concentrations >0.75 M, there was most likely an inhibition of the biocatalyst due to substrate saturation, which has been a well-studied phenomenon for both lipase-mediated esterification and transesterification reactions.^39^ Thus, a 1:3 molar concentration ratio of sorbitol (0.25 M) to vinyl laurate (0.75 M) overall seems more performant. In comparison to the optimized parameters using SDES, the ones obtained in 2M2B are rather more advantageous. Indeed, in comparison, less biocatalyst is needed to achieve considerably higher yields; 50 and 20 g/L Novozym 435 were needed to achieve 28% yield within 48 h in SDES and 97% within 8 h in 2M2B, respectively. In consequence, using 2M2B generates a considerable gain in biocatalyst yield with 8.8 g_product_/g_biocatalyst_ versus 1 g_product_/g_biocatalyst_ when using SDES.

Despite all the advantages, purely performance-wise, of 2M2B over DES, it is important to consider its physical properties, such as flammability and irritancy. Considering the biocompatibility as well as the sustainability of natural DESs, they represent compelling alternative reaction media for production of biomolecules, making them worth further research aiming at industrialized applications.^42,43^

**Figure 4:**
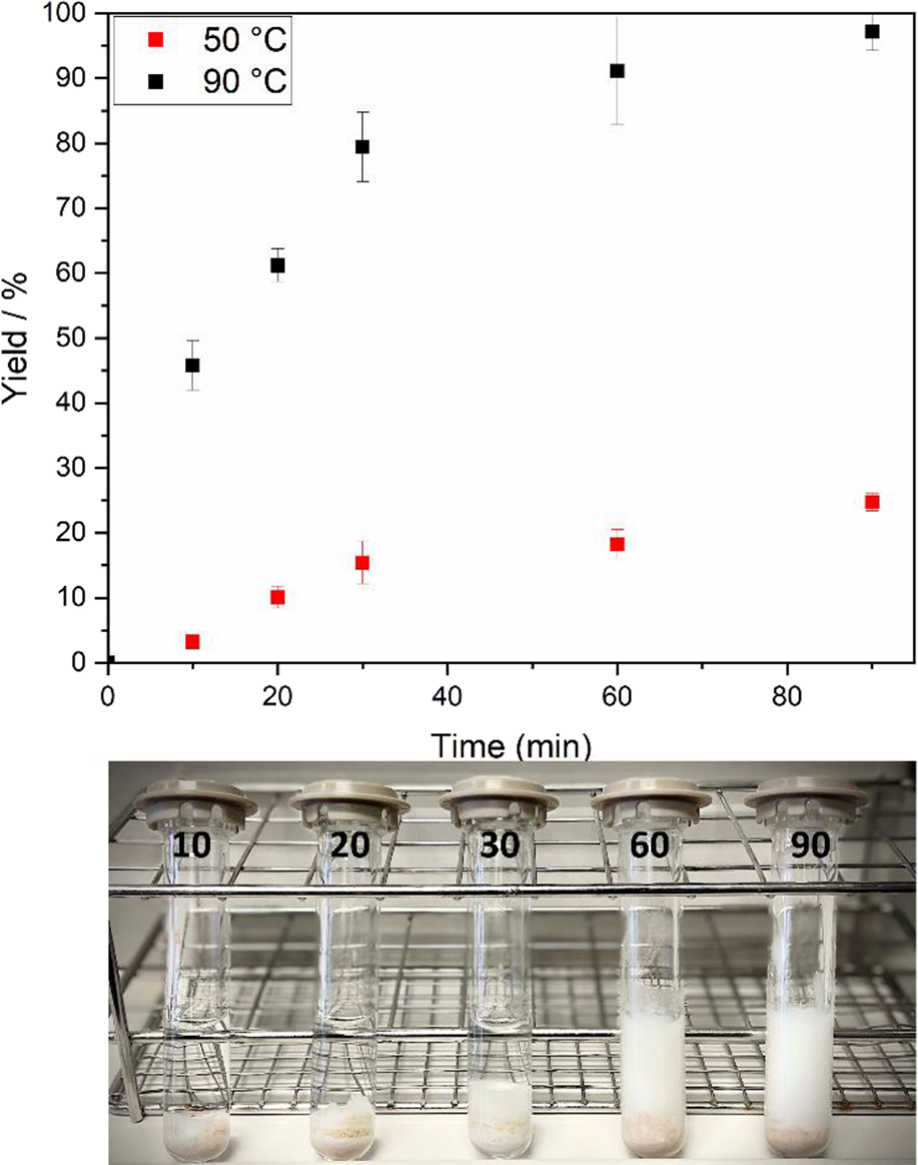
Time course profile for the Novozym 435-catalyzed synthesis of SL at 50 and 90 °C in optimal conditions with CH and corresponding tubes, using magnetic stirring (600 rpm), of the latter at 10, 20, 30, 60, and 90 min in 2M2B.

Using 2M2B as a medium under CH at only 50 °C resulted in an enhanced product concentration, which was reached within a significantly shorter time frame (8 h) (Figure S3) compared to the previously reported SDES system (48 h).^12^ Although these results (Figure 3 and Figure S3) were an already substantial improvement, we aimed at enhancing even further the performances of our process, as displayed in Figure 4. In correlation to a report by Nieto et al., in which reaction equilibrium with xylityl laurate was reached within 90 min in solvent-free and ultrasound-assisted conditions, we decided to investigate the performance of our optimized process assisted by CH within a similar time frame and significantly higher temperature.^44^ By carrying the optimized reaction at 90 °C under CH, an equally high yield (98%) of SL was reached within 90 min of reaction equating to 118.0 ± 3.6 g/(L·h) space-time yield. This represents a several fold improvement from the initial process using DES as well as 2M2B in addition to being a substantial and a significant improvement according to modern bioprocess intensification standards.^45,46^

After 90 min of reaction at 90 °C, formation of a white precipitate was observed in the upper phase when letting the product-supersaturated mixture (176 ± 6 g/L SL) cool down to room temperature, which emphasizes promising purification and overall downstream processing strategies to recover highly pure SL. The precipitate was easily scooped out and analyzed via HPLC-ESLD to be identified as 93% pure SL with traces of unreacted sorbitol, which is beneficial in view of industrial-size production (Figure S4). The presence of traces of unreacted sorbitol in the final mixture might be still acceptable for technical applications but could be problematic to assess surfactant performance. Further purification by preparative chromatography and full structural characterization have been demonstrated in previous studies.^47−49^

### Effect of Temperature and Stirring under Microwave Heating in 2M2B

**Figure 5:**
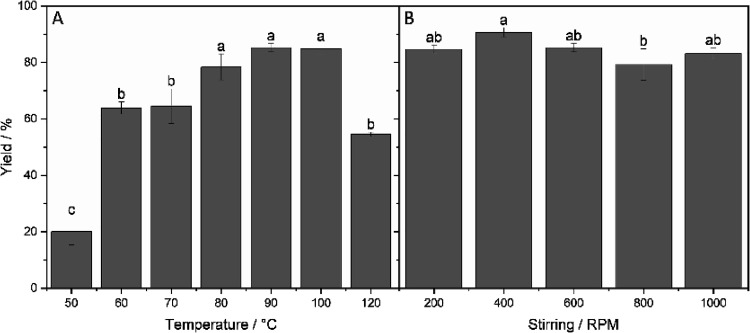
Effect of (A) temperature and (B) stirring on yields with MW heating in 2M2B.

To successfully apply microwave technology to a (bio)chemical process, solvents with strong microwave absorption properties are necessary. The two main solvent properties accountable for the microwave dielectric heating response are the intrinsic polarity and the ionic behavior. Additional desirable physicochemical properties are the high thermal stability and low vapor pressure of the media. Microwave-assisted processes using DESs as solvent media can offer several advantages such as low energy consumption, fast processing times, and high dissolving properties.^50^ Thus, in our case, both SDES and 2M2B were suitable for the application of microwave irradiation as they respond to these requirements. To assess suitability of MW heating for the process intensification of enzymatic SL production, further parameters internal to the MW reactor were investigated such as temperature and stirring speed as it was similarly done for SDES in a previous report of our group.^12^ While Figure 5B does not allow us to identify any trend regarding the influence of stirring speed on yield, Figure 5A gives a rather clear overview of the resilience that the widely used biocatalyst Novozym 435 offers.

Consistently with CH, higher temperatures were also applicable and accelerated the reaction using MW. Indeed, only when a temperature above 100 °C is reached can a decrease in yield be observed. This trend could be naturally explained by potential inactivation of the enzyme due to leakage coupled to denaturation of the enzyme′s tertiary structure upon temperatures exceeding 100 °C,^6^ or simply as the enzyme being less active when free from its carrier, which has been demonstrated several times with lipases since immobilization increases their performance in organic solvents.^51^ Nonetheless, it is noteworthy for a protein to retain >50% catalytic activity under these relatively harsher conditions, most likely owing to the synergy between the carrier (Lewatit VP OC 1600) and the catalytic material (CalB), which is noncovalently immobilized onto its surface. In addition, the biocatalyst showed mechanical resistance to the magnetic stirring as the beads could be easily recovered and reused for further batch experiments, which is a great advantage when aiming for industrial applications.

For all subsequent tests, stirring speed was chosen to be 400 rpm based on Figure 5B combined with the observation of apparent homogeneity made through the built-in camera system inside the microwave (see Figure S5).

### Performance Comparisons between Organic and Deep Eutectic Solvent Systems

In Table 1, we compared the performance obtained with both SDES and 2M2B at two different temperatures, namely, 50 and 90 °C, carried out by either a conventional or microwave heating for the local optimization of the process.

**Table 1:** Impact of Heating, Solvent System, and Temperatures on Process Performances using Novozym 435 for the Biocatalyzed Production of SL at the Tube Scale

heating	solvent system	temperature (°C)	reaction time (h)	space-time yield (g/(L·h))^a^^,^^b^	yield (%)^a^^,^^b^
MW	SDES	50	4	7.5 ± 2.1	7 ± 2
		90	4	16.1 ± 0.6	13 ± <1
	2M2B	50	1.5	29.9 ± 10.2	25 ± 8
		90	1.5	109.2 ± 1.9	90 ± 1
CH	SDES	50^11^	48	1.0 ± 0.1	28 ± 2
		90	1.5	na	na
	2M2B	50	1.5	30.1 ± 0.5	25 ± 1
		90	1.5	118.0 ± 3.6	97 ± 3

a *n* = 3.

b Calculated from eqs 1–3 for the respective reaction media. na, no activity.

Based on these results, it can be confirmed that, unexpectedly, the beneficial effects of MW heating such as an enhanced collision of molecules and increased entropy in the system that would have allowed a higher lipase activity were not as pronounced as expected in the case of 2M2B. In contrast, a significant increase in productivity was achieved for SDES in the microwave. At 50 °C, the space-time yield was improved 7-fold (from 1.0 ± 0.1 g/(L·h) in CH to 7.5 ± 2.1 g/(L·h) in MW). Increasing the reaction temperature to 90 °C led to a further increase in the space-time yield to 16.1 ± 0.6 g/(L·h). Thus, a space-time yield greater than 10 g/(L·h) could be achieved and the process can be considered as economically interesting for production of bulk chemicals according to Lima-Ramos et al.^52^ In addition, no activity was observed at 90 °C in CH after 90 min of reaction.

As mentioned earlier, there is a direct absorption of energy by the functional groups having ionic conductivity or a dipole rotational effect under microwave irradiation. This absorption increases the reactivity of the chemical functions with surrounding reactants compared to convective incubation at the same temperature.^53^ Moreover, as there are always traces of residual water in immobilized enzyme beads, which gets rapidly heated due to thermal effects (dipolar and charge polarization) and specific effects (purely nonthermal), this leads to an improved enzyme activity.^54^ Those effects were more pronounced in this study for the highly viscous SDES system as the 2M2B system already showed nearly full conversion within 90 min at 90 °C in CH.

A similar study has shown that monomode reactors have been successfully used for the synthesis of compounds structurally comparable to SL, such as isoamyl myristate, thus formerly motivating our investigations.^55^

Besides closed MW reactors with magnetic stirring, baffled MW reactors also exist, which thus led to a more efficient mixing. One research axis could be therefore to compare the performance of a closed MW reactor, like the one used in this study, to an open one with seemingly better stirring and higher mass transfer coefficient to improve the reaction in SDES even further.^26^

### Enzyme Recycling in MW and CH

**Figure 6:**
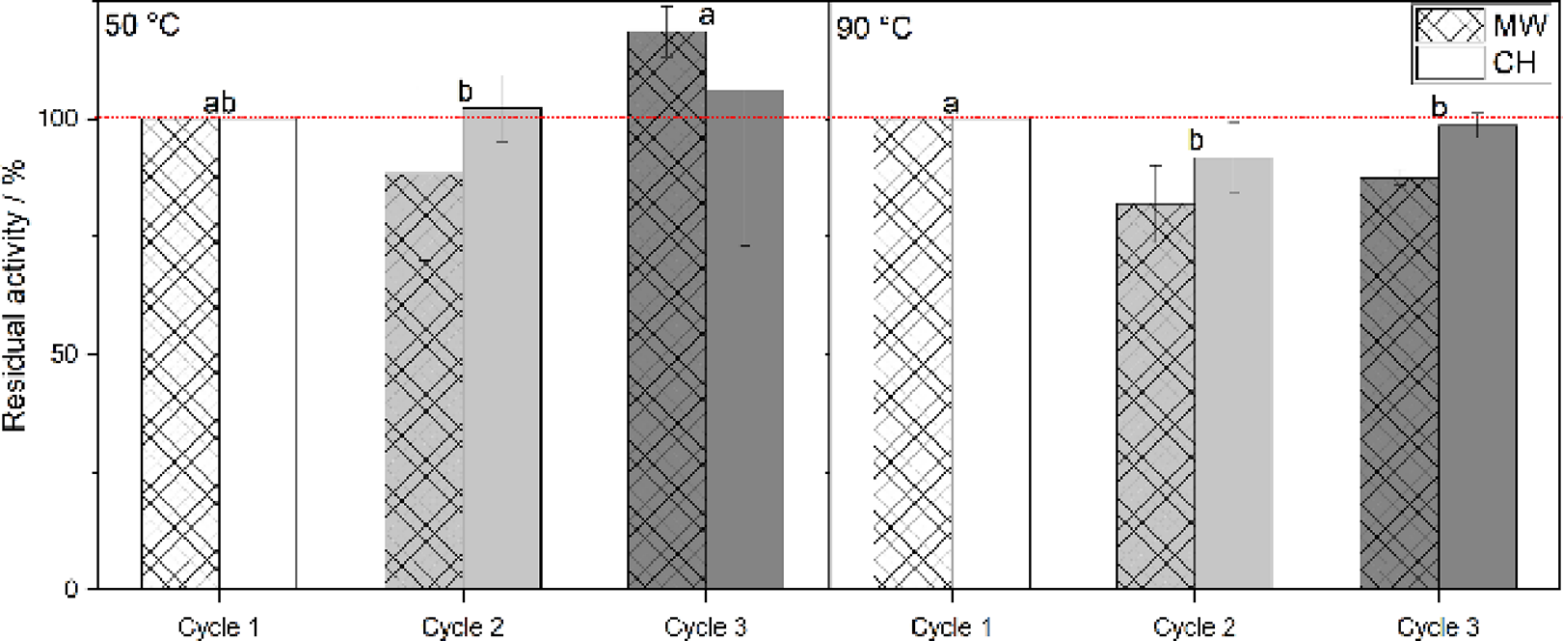
Influence of temperature and heating systems on the reusability of the biocatalyst over three batch reaction cycles of 90 min in 2M2B each.

Novozym 435 was reused for three cycles in 2M2B, each cycle with a total duration of 90 min. Post reaction, the beads were washed with warm reaction media, so the biocatalyst was straightforwardly recovered and subsequently reused. Consequently, the influences of both temperature and heating system on the biocatalyst were compared and are displayed on Figure 6. After three cycles of effective use, 4.5 h in total, it seems that the biocatalyst retained most of its activity at 50 °C, which is greatly valuable for industrial applications as it can drastically increase cost-effectiveness of the process. However, it seems that carrying the reaction at 90 °C has, comparatively, a slightly more deleterious impact on the enzyme′s stability as it loses about 10 to 20% of its performance after the first cycle. This could be explained by a greater leaking of the enzyme at higher temperatures coupled to a greater destabilization of the enzyme structure such as protein unfolding or tertiary structure modification. In follow-up studies, effects on the tertiary or quaternary structures of the enzymes could be assessed via cryogenic electron microscopy (cyro-EM).

As discussed previously, weak motion in the mixtures could potentially lead to the formation of uneven heating spots and temperature gradients, which also results in enzyme denaturation. Heat provided by microwave irradiation induced potential disruption and breakdown of weak ionic and hydrogen bonds.

Liu and Duan reported that Novozym 435 can be recycled using filtration,^56^ which was also demonstrated up to three cycles in this work. The catalytic activity of the enzyme in 2M2B remained high with a rising number of cycles. Therefore, it was demonstrated that Novozym 435 is reusable under microwave conditions for our reaction, which is a good basis for designing further improved industrial processes. Microwave technology can even be applied in continuous flow processing, in which enzyme stability is a crucial requirement. Indeed, in an aim toward industrialization, Morschhäuser et al. demonstrated the first general purpose industrial scale continuous flow microwave reactor, which allowed the safe and highly energy efficient processing of organic reaction or solvent-free mixtures under high temperature/high pressure conditions. The authors noted that the reactor can also be used at moderate temperatures that are relevant for the synthesis of pharmaceutical intermediates, which could be transferable to biocatalysis.^57^ It is worth mentioning that thermogravimetric analysis (TGA) could help investigate the water content of the bead-shaped biocatalytic formulation before and after reaction as well as in between reuse cycles to investigate the consistency of the noncatalytic carrier material.

### Biocatalyzed Solvent-Free Reaction

As of late, solvent-free systems for synthesis have gained popularity due to increased interest in green chemistry principles and metrics.^58^ By design, biotechnology tends to follow these concepts; thus, the rationale to connect biocatalysis and solvent-free systems can be easily understood. In that aim, few papers have made the bridge into that niche topic while displaying rather convincing results.

**Figure 7:**
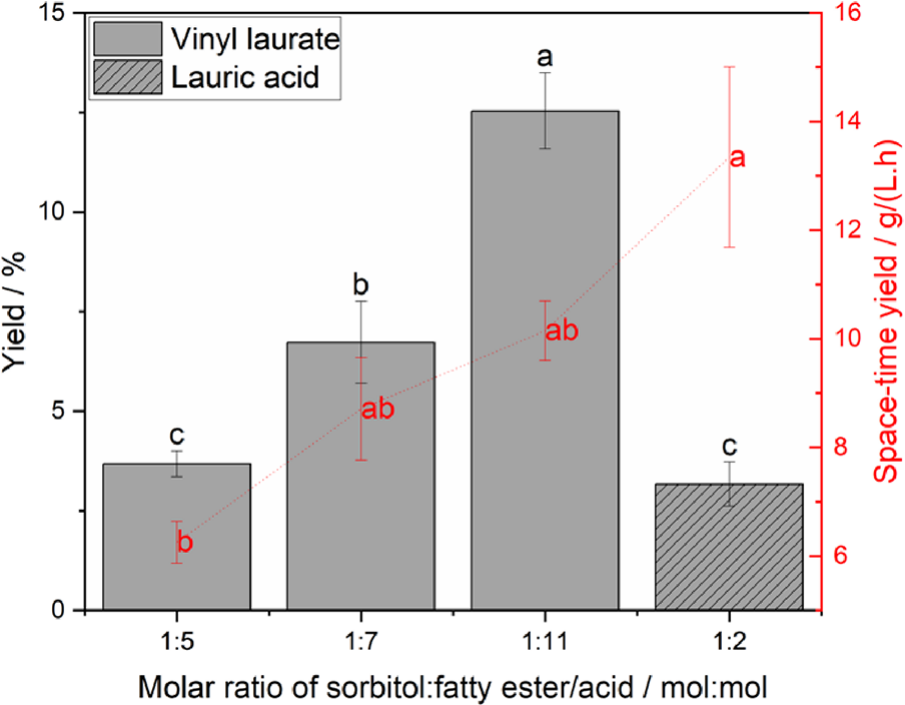
Influence of molar ratio (sorbitol to fatty ester or acid) on yield and space-time yield of SL production using a solvent-free system after 90 min of reaction in MW.

We describe in this subsection, the initial proof of concept for a solvent-free, biocatalyzed, and intensified production of sugar alcohol esters mediated by microwaves. Both vinyl laurate and lauric acid were tested in various molar ratios. Vinyl laurate was, however, a more suitable substrate as it is a liquid at room temperature and thus displays the advantageous characteristics of an adjuvant. In contrast, lauric acid could only be used in a very narrow range of molar ratios. Indeed, anything above or below a 1:2 molar ratio of sorbitol to lauric acid resulted in an unsuccessful synthesis. Second, despite being a liquid above 44.2 °C, which allowed the reaction to proceed to a certain degree, the end mixture would almost instantly crystallize at the contact of colder air because of the physicochemistry of lauric acid. This rendered the extraction and analysis of the target compound challenging and questioned the relevance of such a process at a scale. In Figure 7, we present the performance of this newly developed system as a function of both yield (% conversion of sorbitol) and space-time yield (g/(L·h)). A clear trend can be identified as a ratio 1:11 presented significantly better results in terms of yields (13 ± 1%) when vinyl laurate was used as a substrate compared to the other molar ratios and when lauric acid was used as a building block. Comparatively, the system using lauric acid allowed us to reach a slightly higher space-time yield (13.3 ± 2.3 g/(L·h)) while the yield was drastically lower, which can be explained by a higher concentration of sorbitol for that specific case. Indeed, with the latter, a mole ratio of 1:2 sorbitol and lauric acid had to be used to see lipase activity. Oppositely, this same mole ratio did not yield any results when using vinyl laurate. It is important to note that in our solvent-free method, all amounts of substances, and thereby mole ratios, for the sugar alcohol and fatty ester or acid needed to be adjusted to conserve a minimal operational volume equal to 3 mL, which corresponds to the lowest volume limit allowed for safe use in our microwave setting. As a result of the higher solid loading of lauric acid in the system, it was observed that the bulk of the reaction mixture was hardly stirred and homogenic. Consequently, a very low conversion yield was obtained.

The solvent-free approach is a promising strategy for intensified sustainable processes as the operational volume can be reduced while conserving substantially high productivities. However, it still has significantly lower productivities and yields in comparison to the reaction in 2M2B and thus further optimization in that direction is necessary. It is crucial to note that only the MW reactor made the solvent-free system work within a 90 min time frame as no reaction was observed in CH with identical reaction parameters, thus making a true advantage of using MW over CH for mediating solvent-free reactions producing glycolipids.

### Economic Relevance of the Process Intensification

The overall goal of process intensification is to achieve an economically and ecologically feasible process. There are some parameters that provide guidance in assessment of the current development status, which can be correlated with both economic and ecological requirements. The space-time yield is a widely used metric determining the capital costs and energy requirements to achieve a given productivity. For the production of bulk chemicals, the threshold for the space-time yield to achieve a feasible process is 10 g/(L·h).^52^

Regarding the results of this study, a 10-fold higher space-time yield than the threshold was achieved for the synthesis of SL in 2M2B. This is complemented by a yield of 97%. The metric yield indicative for the impact of raw material costs has a threshold for bulk chemicals of 95%.^52^ The purification costs are normally estimated using the product concentrations. Here, product precipitation in the investigated process is a great advantage. In summary, these three process metrics indicate therefore a promising intensification of SL production in 2M2B with respect to the feasibility of an industrial process. Comparing the productivity reached for SL in 2M2B (118.0 ± 3.6 g/(L·h)) with biotechnological productions of commodity chemicals, higher performance was achieved than biotechnological ethanol production (82 g/(L·h)) and almost as high as biotechnological lactic acid production with 150 g/(L·h).^46^

Regarding SL production in alternative reaction media, i.e., SDES in combination with MW heating, as well as the solvent-free synthesis, the threshold for the space-time yield for bulk chemicals was also exceeded (16.1 ± 0.6 g/(L·h) and 13.3 ± 2.3 g/(L·h), respectively). Thus, productivities comparable to the biotechnological production of 1-butanol (10 g/(L·h)), succinate (15 g/(L·h)), and gluconate (19 g/(L·h)) were achieved.^46^ However, the yields of both reactions in highly viscous mixtures need to be improved even further.

The results of this study are in consequence highly promising for the development of biocatalytic SL production toward industrial feasible processes.

## Conclusions

The aim of the study was to investigate the process intensification of enzymatic SL production. Therefore, combinations of two heating methods (conventional and MW) and three solvent systems (organic solvents, DES, and neat) were evaluated. It was shown that increasing the reaction temperature is highly beneficial for local process optimization and that even temperatures of 90 °C are suitable without hampering the enzyme performance of Novozym 435. The results of lipase-catalyzed synthesis of SL previously obtained in DES with CH were compared to the results of this study carried out in organic solvents. Synthesis in low viscous 2M2B was more profitable in terms of production yields and reaction time compared to highly viscous DES as the reaction media. The influence of the heating method on glycolipid production was investigated by using MW irradiation for both the low-viscosity organic solvent and the high-viscosity DES. MW technology did not yield process intensification in low-viscosity solvents like 2M2B, where proper mass transfer and heat distribution were also reached in CH. In highly viscous reaction mixtures such as DES or neat reaction systems, however, MW heating had substantial effects. The combination of increased temperature and MW heating yielded a 16-fold increase in space-time yields over the initial setup using CH. Therefore, we showed that although heating efficiency is an important parameter in enzymatic reactions, it is less pronounced in reaction systems characterized by already good mass transfer. In highly viscous systems, however, heating efficiency is highly important as it contributes to improved reaction metrics despite limited heat and mass transfer.

The solvent-free syntheses were only made possible by MW technology since it was not successful at all with CH, whereas MW irradiation enabled space-time yields of up to 13.3 ± 2.3 g/(L·h), thus qualifying it as an intensified process. Hence, MW heating is a promising tool for process intensification in highly viscous media and for reducing the number of solvents in biotechnological processes. Moreover, we showed for the first time that enzymes can also be reused over several cycles in MW heating for glycolipid synthesis, which is an important starting point for the development of continuous flow MW processes using enzymes. Even temperatures of 90 °C are suitable without hampering the enzyme performance. In conclusion, this process intensification leads to process metrics that show economic feasibility for the synthesis in 2M2B. Also, for the synthesis in more challenging viscous reaction mixtures, like SDES and solvent-free synthesis, promising space-time yields were achieved, while still further intensification is needed to reach economically feasible yields.

## Supporting Information Available

Calibration curve of SL using a HPLC-ELSD method; chromatogram of in-house-produced SL external standard used for quantification; time course of SL production with CH using 2M2B at 50 °C, chromatogram of raw SL resulting from postreaction precipitation; depiction of the inside of the microwave with a built-in camera (PDF)

## ABBREVIATIONS

DES(s): deep eutectic solvent(s)
2M2B: 2-methyl-2-butanol
MW: microwave
CH: conventional heating
SL: sorbitol-6-*O*-laurate
SDES: sorbitol-based deep eutectic solvent
EL: ethyl-L-lactate
MeCN: acetonitrile
MEK: methyl ethyl ketone
ACE: acetone
EtOH: ethanol
ELSD: evaporative light scattering detector
CalB: *Candida antarctica* lipase B
Log P: logarithm of partition coefficient

## References

[ref1] Farias C. B. B.; et al. Production of green surfactants: Market prospects. Electron. J. Biotechnol. 2021, 51, 28–39. 10.1016/j.ejbt.2021.02.002.

[ref2] Victor A. B.; Biermann M.; Hill K.; Raths H.-C.; Saint M.-E. Industrial Surfactant Syntheses and Günter Uphues. in Reactions And Synthesis In Surfactant Systems; CRC Press, 2001.

[ref3] Rebello S.; Asok A. K.; Mundayoor S.; Jisha M. S. Surfactants: Chemistry, Toxicity and Remediation. Environ. Chem. Sustain. World 2013, 277 10.1007/978-3-319-02387-8_5.

[ref4] Grüninger J.; Delavault A.; Ochsenreither K. Enzymatic glycolipid surfactant synthesis from renewables. Process Biochem. 2019, 87, 45–54. 10.1016/j.procbio.2019.09.023.

[ref5] Naughton P. J.; Marchant R.; Naughton V.; Banat I. M. Microbial biosurfactants: current trends and applications in agricultural and biomedical industries. J. Appl. Microbiol. 2019, 127, 12–28. 10.1111/jam.14243. 30828919

[ref6] Ortiz C.; et al. Novozym 435: the “perfect” lipase immobilized biocatalyst?. Catal. Sci. Technol. 2019, 9, 2380–2420. 10.1039/C9CY00415G.

[ref7] Anastas P.; Warner J. Green Chemistry: Theory and Practice. Oxford University Press, 2000.

[ref8] García S.; Zhang L.; Piburn G. W.; Henkelman G.; Humphrey S. M. Microwave Synthesis of Classically Immiscible Rhodium–Silver and Rhodium–Gold Alloy Nanoparticles: Highly Active Hydrogenation Catalysts. ACS Nano 2014, 8, 11512–11521. 10.1021/nn504746u. 25347078

[ref9] Huynh A.; et al. Measurements meet perceptions: rheology–texture–sensory relations when using green, bio-derived emollients in cosmetic emulsions. Int. J. Cosmet. Sci. 2021, 43, 11–19. 10.1111/ics.12661. 32886359

[ref10] Siebenhaller S.; et al. Sustainable enzymatic synthesis of glycolipids in a deep eutectic solvent system. J. Mol. Catal. B Enzym. 2016, 133, S281–S287. 10.1016/j.molcatb.2017.01.015.

[ref11] Pätzold M.; et al. Deep Eutectic Solvents as Efficient Solvents in Biocatalysis. Trends Biotechnol. 2019, 37, 943–959. 10.1016/j.tibtech.2019.03.007. 31000203

[ref12] Delavault A.; et al. Lipase-Catalyzed Production of Sorbitol Laurate in a “2-in-1” Deep Eutectic System: Factors Affecting the Synthesis and Scalability. Molecules 2021, 26, 2759 10.3390/molecules26092759. 34067126 PMC8124474

[ref13] Durand E.; et al. Evaluation of deep eutectic solvents as new media for Candida antarctica B lipase catalyzed reactions. Process Biochem. 2012, 47, 2081–2089. 10.1016/j.procbio.2012.07.027.

[ref14] Francisco M.; van den Bruinhorst A.; Kroon M. C. Low-Transition-Temperature Mixtures (LTTMs): A New Generation of Designer Solvents. Angew. Chem., Int. Ed. 2013, 52, 3074–3085. 10.1002/anie.201207548. 23401138

[ref15] Kottaras P.; Koulianos M.; Makris D. Low-Transition Temperature Mixtures (LTTMs) Made of Bioorganic Molecules: Enhanced Extraction of Antioxidant Phenolics from Industrial Cereal Solid Wastes. Recycling 2017, 2, 3 10.3390/recycling2010003.

[ref16] Rodríguez N. R.; González A. S. B.; Tijssen P. M. A.; Kroon M. C. Low transition temperature mixtures (LTTMs) as novel entrainers in extractive distillation. Fluid Phase Equilib. 2015, 385, 72–78. 10.1016/j.fluid.2014.10.044.

[ref17] Tang B.; Row K. H. Recent developments in deep eutectic solvents in chemical sciences. Monatshefte Für Chem. - Chem. Mon. 2013, 144, 1427–1454. 10.1007/s00706-013-1050-3.

[ref18] Keil F. J. Process intensification. Rev. Chem. Eng. 2018, 34, 135–200. 10.1515/revce-2017-0085.

[ref19] Priecel P.; Lopez-Sanchez J. A. Advantages and Limitations of Microwave Reactors: From Chemical Synthesis to the Catalytic Valorization of Biobased Chemicals. ACS Sustain. Chem. Eng. 2019, 7, 3–21. 10.1021/acssuschemeng.8b03286.

[ref20] Rathi A. K.; Gawande M. B.; Zboril R.; Varma R. S. Microwave-assisted synthesis – Catalytic applications in aqueous media. Coord. Chem. Rev. 2015, 291, 68–94. 10.1016/j.ccr.2015.01.011.

[ref21] Khan N. R.; Rathod V. K. Microwave assisted enzymatic synthesis of speciality esters: A mini - review. Process Biochem. 2018, 75, 89–98. 10.1016/j.procbio.2018.08.019.

[ref22] Yadav G. D.; Lathi P. S. Synergism between microwave and enzyme catalysis in intensification of reactions and selectivities: transesterification of methyl acetoacetate with alcohols. J. Mol. Catal. Chem. 2004, 223, 51–56. 10.1016/j.molcata.2003.09.050.

[ref23] Yadav G. D.; Lathi P. S. Synergism of Microwaves and Immobilized Enzyme Catalysis in Synthesis of Adipic Acid Esters in Nonaqueous Media. Synth. Commun. 2005, 35, 1699–1705. 10.1081/SCC-200061687.

[ref24] Yadav G. D.; Sajgure A. D. Synergism of microwave irradiation and enzyme catalysis in synthesis of isoniazid. J. Chem. Technol. Biotechnol. 2007, 82, 964–970. 10.1002/jctb.1738.

[ref25] Kuo C.-H.; Chen H.-H.; Chen J.-H.; Liu Y.-C.; Shieh C.-J. High Yield of Wax Ester Synthesized from Cetyl Alcohol and Octanoic Acid by Lipozyme RMIM and Novozym 435. Int. J. Mol. Sci. 2012, 13, 11694–11704. 10.3390/ijms130911694. 23109878 PMC3472770

[ref26] Yadav G. D.; Borkar I. V. Kinetic and Mechanistic Investigation of Microwave-Assisted Lipase Catalyzed Synthesis of Citronellyl Acetate. Ind. Eng. Chem. Res. 2009, 48, 7915–7922. 10.1021/ie800591c.

[ref27] Dai Y.; Witkamp G.-J.; Verpoorte R.; Choi Y. H. Tailoring properties of natural deep eutectic solvents with water to facilitate their applications. Food Chem. 2015, 187, 14–19. 10.1016/j.foodchem.2015.03.123. 25976992

[ref28] Hayyan A.; et al. Glucose-based deep eutectic solvents: Physical properties. J. Mol. Liq. 2013, 178, 137–141. 10.1016/j.molliq.2012.11.025.

[ref29] Flores M. V.; Halling P. J. Full model for reversible kinetics of lipase-catalyzed sugar–ester synthesis in 2-methyl 2-butanol. Biotechnol. Bioeng. 2002, 78, 795–801. 10.1002/bit.10260. 12001171

[ref30] Khaled N.; Montet D.; Pina M.; Graille J. Fructose oleate synthesis in a fixed catalyst bed reactor. Biotechnol. Lett. 1991, 13, 167–172. 10.1007/BF01025812.

[ref31] Ijardar S. P.; Singh V.; Gardas R. L. Revisiting the Physicochemical Properties and Applications of Deep Eutectic Solvents. Molecules 2022, 27, 1368 10.3390/molecules27041368. 35209161 PMC8877072

[ref32] Laane C.; Boeren S.; Vos K.; Veeger C. Rules for optimization of biocatalysis in organic solvents. Biotechnol. Bioeng. 1987, 30, 81–87. 10.1002/bit.260300112. 18576586

[ref33] Nian B.; Cao C.; Liu Y. How Candida antarctica lipase B can be activated in natural deep eutectic solvents: experimental and molecular dynamics studies. J. Chem. Technol. Biotechnol. 2020, 95, 86–93. 10.1002/jctb.6209.

[ref34] Gawas S. D.; Jadhav S. V.; Rathod V. K. Solvent Free Lipase Catalysed Synthesis of Ethyl Laurate: Optimization and Kinetic Studies. Appl. Biochem. Biotechnol. 2016, 180, 1428–1445. 10.1007/s12010-016-2177-6. 27470112

[ref35] Jaiswal K. S.; Rathod V. K. Microwave-assisted synthesis of ethyl laurate using immobilized lipase: Optimization, mechanism and thermodynamic studies. J. Indian Chem. Soc. 2021, 98, 100020 10.1016/j.jics.2021.100020.

[ref36] Jahangiri A.; et al. Hydrophilization of bixin by lipase-catalyzed transesterification with sorbitol. Food Chem. 2018, 268, 203–209. 10.1016/j.foodchem.2018.06.085. 30064749

[ref37] Li C.; Tan T.; Zhang H.; Feng W. Analysis of the conformational stability and activity of Candida antarctica lipase B in organic solvents: insight from molecular dynamics and quantum mechanics/simulations. J. Biol. Chem. 2010, 285, 28434–28441. 10.1074/jbc.M110.136200. 20601697 PMC2937868

[ref38] Elgharbawy A. A.; et al. Shedding Light on Lipase Stability in Natural Deep Eutectic Solvents. Chem. Biochem. Eng. Q. 2018, 32, 359–370. 10.15255/CABEQ.2018.1335.

[ref39] Arcens D.; Grau E.; Grelier S.; Cramail H.; Peruch F. Impact of Fatty Acid Structure on CALB-Catalyzed Esterification of Glucose. Eur. J. Lipid Sci. Technol. 2020, 122, 1900294 10.1002/ejlt.201900294.

[ref40] Alder C. M.; et al. Updating and further expanding GSK’s solvent sustainability guide. Green Chem. 2016, 18, 3879–3890. 10.1039/C6GC00611F.

[ref41] Zhao K.-H.; et al. Enzymatic Synthesis of Glucose-Based Fatty Acid Esters in Bisolvent Systems Containing Ionic Liquids or Deep Eutectic Solvents. Molecules 2016, 21, 1294 10.3390/molecules21101294. 27689970 PMC6273948

[ref42] Espino M.; de los Ángeles Fernández M.; Gomez F. J. V.; Silva M. F. Natural designer solvents for greening analytical chemistry. TrAC Trends Anal. Chem. 2016, 76, 126–136. 10.1016/j.trac.2015.11.006.

[ref43] Gomez F. J. V.; Espino M.; de Los Angeles Fernandez M.; Raba J.; Silva M. F. Enhanced electrochemical detection of quercetin by Natural Deep Eutectic Solvents. Anal. Chim. Acta 2016, 936, 91–96. 10.1016/j.aca.2016.07.022. 27566343

[ref44] Nieto S.; Villa R.; Donaire A.; Lozano P. Ultrasound-assisted enzymatic synthesis of xylitol fatty acid esters in solvent-free conditions. Ultrason. Sonochem. 2021, 105606 10.1016/j.ultsonch.2021.105606. 34058635 PMC8170488

[ref45] Boodhoo K. V. K.; Flickinger M. C.; Woodley J. M.; Emanuelsson E. A. C. Bioprocess intensification: A route to efficient and sustainable biocatalytic transformations for the future. Chem. Eng. Process. - Process Intensif. 2022, 172, 108793 10.1016/j.cep.2022.108793.

[ref46] Straathof A. J. J. Transformation of Biomass into Commodity Chemicals Using Enzymes or Cells. Chem. Rev. 2014, 114, 1871–1908. 10.1021/cr400309c. 23987659

[ref47] Iyer R. The Problem of Purity in Evaluating Surfactant Performance. Tenside Surfactants Deterg. 2005, 42, 336–341. 10.3139/113.100276.

[ref48] Delavault A.; et al. Microwave-Assisted One-Pot Lipid Extraction and Glycolipid Production from Oleaginous Yeast Saitozyma podzolica in Sugar Alcohol-Based Media. Molecules 2021, 16, 470 10.3390/molecules26020470. PMC7829979 33477445

[ref49] Hollenbach R.; Ochsenreither K.; Syldatk C. Enzymatic Synthesis of Glucose Monodecanoate in a Hydrophobic Deep Eutectic Solvent. Int. J. Mol. Sci. 2020, 21, 4342 10.3390/ijms21124342. 32570792 PMC7352255

[ref50] González-Rivera J.; et al. Insights into microwave heating response and thermal decomposition behavior of deep eutectic solvents. J. Mol. Liq. 2020, 300, 112357 10.1016/j.molliq.2019.112357.

[ref51] Rueda N.; et al. Improved performance of lipases immobilized on heterofunctional octyl-glyoxyl agarose beads. RSC Adv. 2015, 5, 11212–11222. 10.1039/C4RA13338B.

[ref52] Lima-Ramos J.; Tufvesson P.; Woodley J. M. Application of environmental and economic metrics to guide the development of biocatalytic processes. Green Process. Synth. 2014, 3, 195–213. 10.1515/gps-2013-0094.

[ref53] Sontakke J. B.; Yadav G. D. Optimization and kinetic modeling of lipase catalyzed enantioselective N-acetylation of (±)-1-phenylethylamine under microwave irradiation. J. Chem. Technol. Biotechnol. 2011, 86, 739–748. 10.1002/jctb.2582.

[ref54] Loupy A.; Perreux L.; Liagre M.; Burle K.; Moneuse M. Reactivity and selectivity under microwaves in organic chemistry. Relation with medium effects and reaction mechanisms. Pure Appl. Chem. 2001, 73, 161–166. 10.1351/pac200173010161.

[ref55] Yadav G. D.; Thorat P. A. Microwave assisted lipase catalyzed synthesis of isoamyl myristate in solvent-free system. J. Mol. Catal. B Enzym. 2012, 83, 16–22. 10.1016/j.molcatb.2012.06.011.

[ref56] Liu W.; Duan F. Lipase-catalyzed transesterification of epoxidized soybean oil to prepare epoxy methyl esters. Grasas Aceites 2018, 69, e247–e247. 10.3989/gya.1103172. PMC9079759 35541221

[ref57] Morschhäuser R.; Krull M.; Kayser C.; Boberski C.; Bierbaum R.; Püschner P. A.; Glasnov T. N.; Kappe C. O.; et al. Microwave-assisted continuous flow synthesis on industrial scale. Green Process. Synth. 2012, 1, 281–290. 10.1515/gps-2012-0032.

[ref58] Sheldon R. A. Metrics of Green Chemistry and Sustainability: Past, Present, and Future. ACS Sustain. Chem. Eng. 2018, 6, 32–48. 10.1021/acssuschemeng.7b03505.

