# Supplementary material for: Intensification of Enzymatic Sorbityl Laurate Production in Dissolved and Neat Systems under Conventional and Microwave Heating
Source: ACS Omega. 2024 Apr 1;9(15):17163–73. doi: 10.1021/acsomega.3c10004 (PMC11024949; doi:10.1021/acsomega.3c10004)
Supplement: Supplementary file 1 — ao3c10004_si_001.pdf [file ao3c10004_si_001.pdf]

Supporting Information

Intensification of enzymatic sorbityl laurate production in dissolved and neat systems under conventional and microwave heating

André Delavault†[1], Oleksandra Opochenska[1], Sonja Schönrock[1], Rebecca Hollenbach[2], Katrin Ochsenreither[2] and Christoph Syldatk[1]*

[1]Technical Biology, Institute of Process Engineering in Life Sciences II, Karlsruhe Institute of Technology, Karlsruhe, Germany.

[2]Biotechnologische Konversion, Technikum Laubholz GmbH, Göppingen, Germany.

**corresponding author: andre.delavault@kit.edu*

†present address: Institute for Biological Interfaces 1, Karlsruhe Institute of Technology, Eggenstein-Leopoldshafen, Germany.

Number of pages: 6

Number of figures: 5

Number of tables: 0

Content

Figure S1…………….... ……………………………………………………...……...………S4

Figure S2. ………………………………………………………….....……...………S4

Figure S3. ……………………………………………...……...…………...………S5

Figure S4. …………………………………………………..……………...………S6

Figure S5. ……………………………………………...…………………...……S6

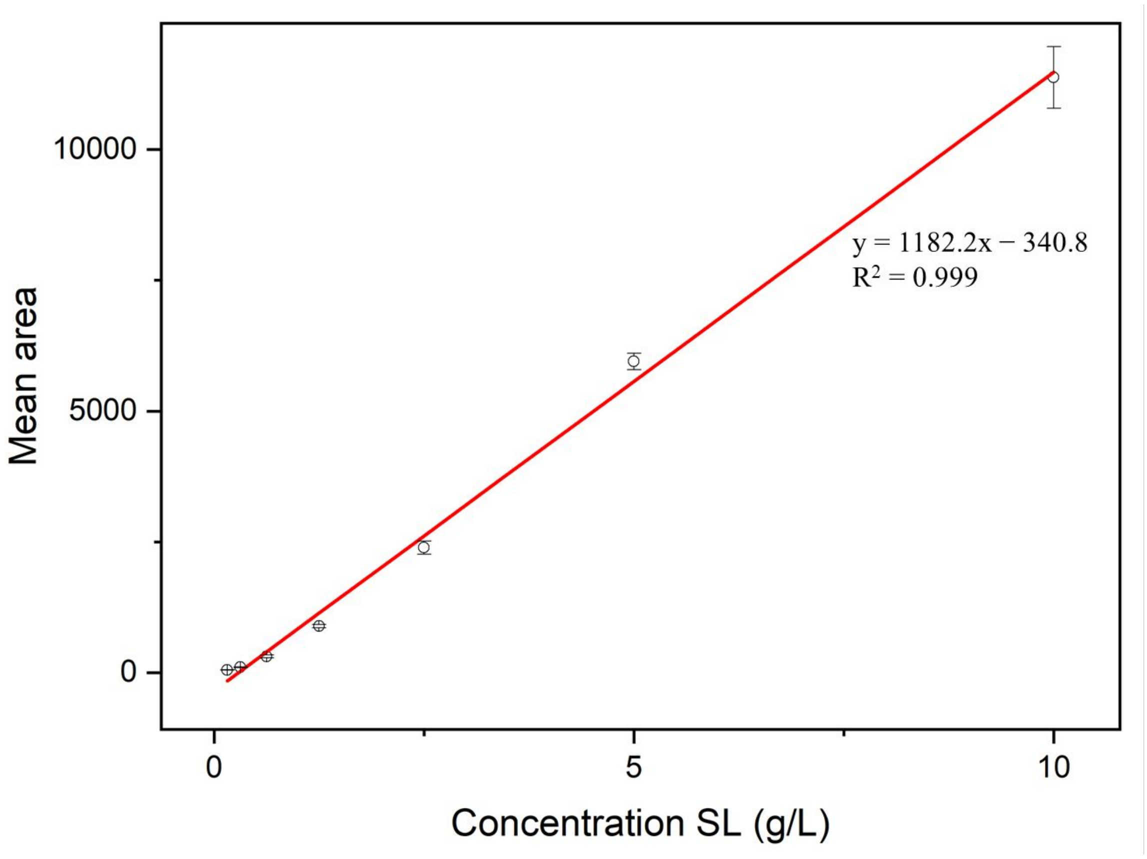

Figure S1. Linear regression of the straight-line calibration curve for the quantification of SL concentration post-reaction.

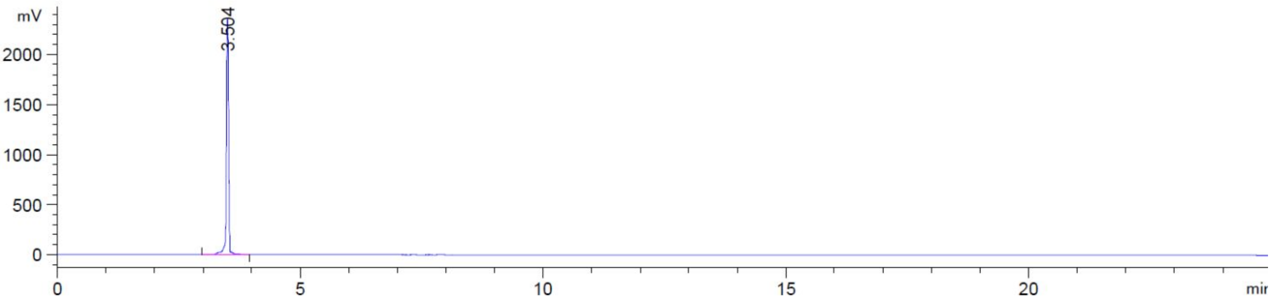

Figure S2. In-house produced SL standard used for calibration and quantification injected at 17.5 g/L.

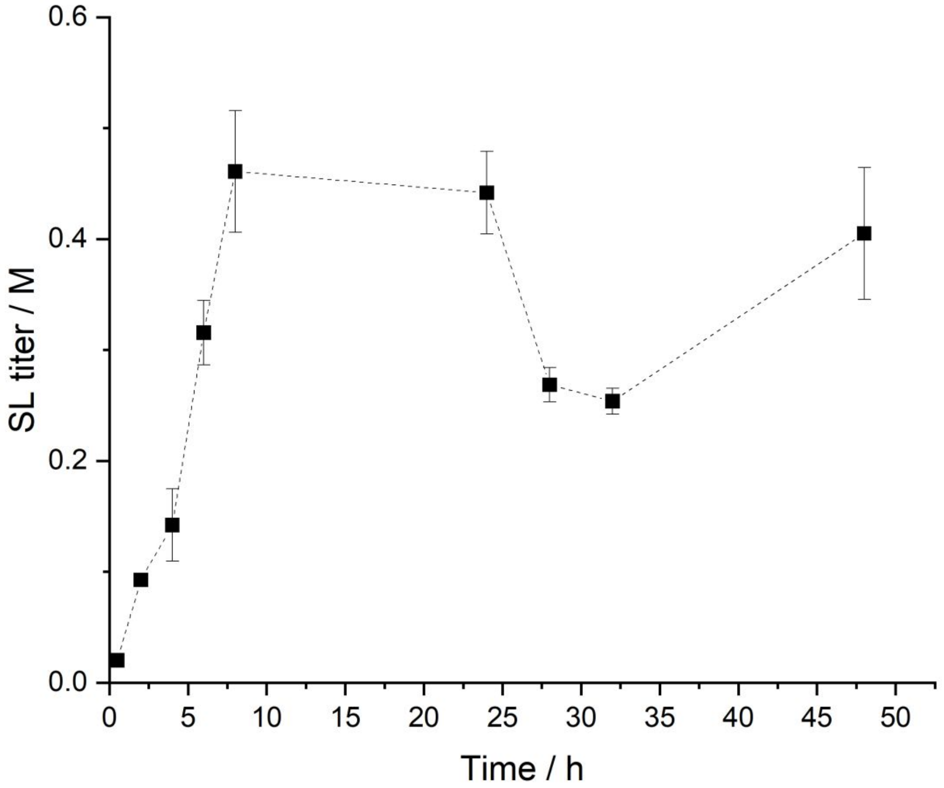

***Figure S3*.** Time course of the SL production synthesis under optimized conditions at 50°C with CH using 2M2B as reaction media. Conditions used were as such: 0.25 M sorbitol, 0.75 M vinyl laurate and 20 g/L of Novozym 435.

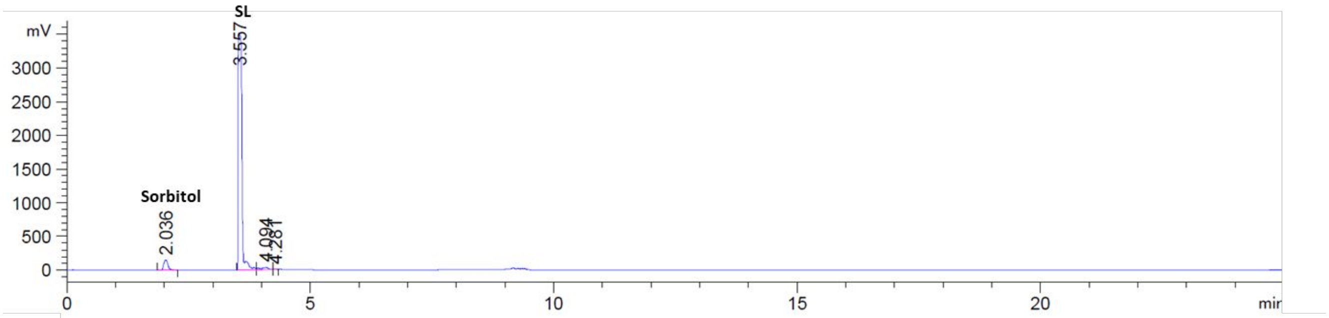

Signal 1: ELS1 A, Signal Voltage

Peak #	RetTime [min]	Type	Width [min]	Area [mV*s]	Height [mV]	Area %
1	2.036	BV	0.0975	891.38776	149.73743	4.5291
2	3.557	BV	0.0850	1.82941e4	3498.39331	92.9512
3	4.094	VV	0.1761	441.65250	33.88270	2.2440
4	4.281	VV	0.0710	54.26323	10.73552	0.2757
Totals :				1.96814e4	3692.74896	

Figure S4. Chromatography diagram resulting from the injection of the precipitate scooped out after 90 minutes reaction carried in 2M2B using CH. Concentration of the precipitate at 26.7 g/L in $CHCl_3$:MeOH (7:3). Peak at 3.557 min has been fully characterized as targeted monoester sorbityl-6-O-laurate (Delavault et al. 2021).

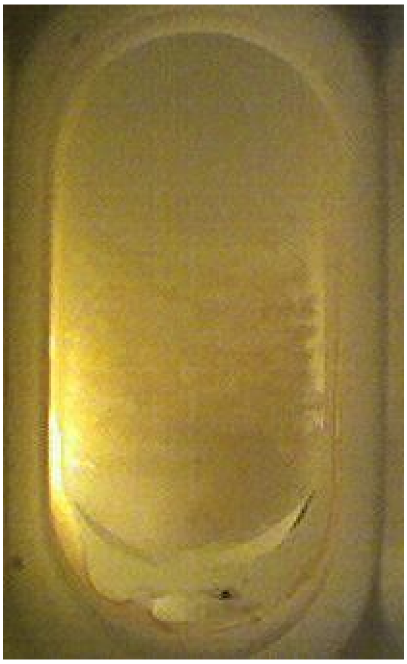

Figure S5. Depiction of the reaction inside the Anton-Paar microwave Monowave 400 using the built-in camera system when performing SL synthesis at 90 °C.
